# From by-Product to Unconventional Vegetable: Preliminary Evaluation of Fresh Fava Hulls Highlights Richness in L-Dopa and Low Content of Anti-Nutritional Factor

**DOI:** 10.3390/foods9020159

**Published:** 2020-02-07

**Authors:** Massimiliano Renna, Francesca De Cillis, Beniamino Leoni, Ermelinda Acciardi, Pietro Santamaria

**Affiliations:** 1 Institute of Sciences of Food Production (ISPA), CNR, via Amendola 122/O, 70126 Bari, Italy; 2 Department of Agricultural and Environmental Science, University of Bari Aldo Moro, via Amendola 165/A, 70126 Bari, Italy; beniamino.leoni@uniba.it (B.L.); ermelinda.88@live.it (E.A.); pietro.santamaria@uniba.it (P.S.)

**Keywords:** agrobiodiversity, ascorbic acid, food waste, functional food, local varieties, phenols, plant density, *Vicia faba* L., vicine

## Abstract

Faba bean hulls are a by-product, generated from the processing of beans and usually disposed of as waste, utilized in some recipes of Italian traditional cuisine. In this research, a quality evaluation of faba hulls in six genotypes (four local varieties—‘Cegliese’, ‘Iambola’, ‘San Francesco’ and ‘FV5′—and two commercial ones—‘Aguadulce supersimonia’ and ‘Extra-early purple’) of faba bean (*Vicia faba* L. var. *major* Harz) for fresh consumption grown with two plant densities (4.16 and 2.08 plants m^−2^) was carried out. For all the measured parameters, the statistical analysis reveals that the interaction between plant density and genotype was not significant. On the other hand, independently of the genotype, the higher the plant’s density the higher was the pods’ yield per unit area, while the average percentage of hulls was of 75% with little differences between genotypes. All genotypes showed a low content of vicine (12.4 mg 100 g^−1^ FW), a well know favism-inducing factor, and a very high phenols content (between 443 and 646 mg 100 g^−1^ FW) and levo-dihydroxy phenylalanine (L-dopa—on average 170 mg 100 g^−1^ FW), used for the treatment of patients affected by Parkinson’s disease. In conclusion, this study highlights the good potential of faba hulls as unconventional vegetable, suggesting its use as a new functional food in the daily diet and also for patients with Parkinson’s disease.

## 1. Introduction

Faba bean (*Vicia faba* L.), also known as broad bean, is widely consumed worldwide for its edible seeds containing a considerable amount of carbohydrates and proteins as well as several other compounds such as fibre, lecithin, choline, minerals and secondary metabolites [1]. Like for other pulses (legumes), faba beans are used in many countries as dry seeds for the high protein content, making them excellent meat substitutes, suitable for vegan and vegetarian diets as well as for reducing the consumption of animal-based food. At the same time, a part of these legumes is aimed at consumption as fresh vegetables, characterized by immature seeds with a higher content of water and lesser protein and starch percentage than dry seeds (mature), but with a greater amount of functional substances such as phenols, chlorophylls, carotenoids and vitamins [2]. For example, De Cillis et al. [3] found a content of ascorbic acid ranging from about 380 to about 700 mg 100 g^−1^ fresh weight (FW) as well as an interesting L-dopa content, a non-protein amino acid usually used for the treatment of Parkinson’s disease and other disease [4]. Unfortunately, it should be considered that nowadays faba beans do not receive a right attention, because they contain some anti-nutritional factors such as vicine and convicine which can cause acute haemolysis phenomena subject with glucose-6-phosphate-dehydrogenase (G6PD) deficiency [5].

An important aspect of legume immature seeds used as vegetables regards their use, since hulls are removed for using seeds as a fresh food or processing them as frozen or canned food [6,7]. As a consequence, a big mass of hulls (about 70% of the total fava bean yield from the field) is generated as by-products, usually disposed of as waste. To this regards, it is interesting to highlight that like for summer squash greens, faba greens and offshoots of globe artichoke [8], also faba hulls can be used as vegetables, instead of being considered as by-products. As a matter of fact, in some areas of Italy boiled faba hulls are traditionally used to prepare some dishes such as first and main courses [9]. The culinary use of faba hulls in Italy, like other vegetable by-products, was made in the past during the periods of poverty and food scarcity, allowing to valorise by-products as “unconventional vegetables” [8]. On the other hand, considering their nutritional traits, nowadays these unconventional vegetables could satisfy the demand for high-quality foods found on ago-biodiversity [10]. It is important to highlight that functional foods contain components, nutrients, or bioactive compounds exerting selective beneficial effects on one or more functions of the organism. In this context, health claims can be considered as important value-added features for consumers and therefore for the food industry, since they can give a competitive advantage to a food product and can differentiate food products in the market. Thus, it could be very interesting to assess the quality as well as healthy compounds of these unconventional vegetables.

Since information in literature on faba hulls are scarce, the aim of this research was to assess some nutritional traits and bioactive compounds of faba hulls for providing an overview related to their potential as a new functional food.

## 2. Materials and Methods 

### 2.1. Cropping Details and Sampling Procedures 

Six genotypes of *Vicia faba L*. were used: four local varieties (Iambola, San Francesco, FV5, Cegliese) and two commercial ones (Extra-early purple and Aguadulce supersimonia). Plants were grown in a 555 m^2^ open field at “La Noria” experimental farm of the Institute of Sciences of Food Production of the Italian National Research Council, located in Mola di Bari (Ba, Italy), 24 m above sea level, 41°03′ N, 17°04′ E. Sowing was carried out in Autumn 2017, by using two plant densities for the production of broad beans for fresh consumption: (i) high (4.16 plants m^−2^—80 cm between rows and 30 cm within the row); (ii) low (2.08 plants m^−2^—160 cm between rows and 30 cm within the row). A split-plot design (plant density in the main plots and the genotypes in the subplots) with three replications; each elemental parcel contained 30 plants for both densities. Plants were grown without fertilization, while a supplemental irrigation was performed only between sowing and emergence of seedling. Four harvests were carried out between April and May 2018 (10/4, 18/4, 24/4, 4/5), according to the progressive ripening of the pods. At each harvest, the pods were weighed and counted to calculate the cumulative yield. Moreover, representative samples from each harvest were used to determine the dry weight. Samples of the 2nd harvest were used for physical and chemical analysis.

### 2.2. Physical Analysis

Colour analysis (CIELab colour scale) and total soluble solids (TSS) content of hulls were carried out by using a colorimeter (CR-400, Konica Minolta, Osaka, Japan) and a refractometer DBR45 (XS Instruments, Carpi, Italy) according to the procedure described by De Cillis et al. [3]. Each measurement was carried out in triplicate by using 30 hulls per sample.

In regards to colour analysis, the colorimeter was calibrated with a standard reference with values L, a* and b* of 97.82, 1.27 and 1.35, respectively. L represents the lightness value; a* represents the red/green index; b* represents the yellow/blue index. Hue angle (h°= tan − 1[b*/a*]) and saturation or chroma (C = [a*^2^ + b*^2^]^1/2^) were then calculated from the primary L, a* and b* readings.

Regarding TSS content the analysis were carried out on hull’s liquid extract and results were expressed in °Brix.

For the dry weight (DW) determination, samples were maintained in an air oven at 105 °C until constant weight. 

### 2.3. Total Titratable Acidity, pH and Crude Protein Content

Titratable acidity (TA) and pH were measured according to the procedure described by De Cillis et al. [3] by using a pH meter Model 507 (Crison, Milan, Italy), while the crude protein content (N × 6.25) was determined according to the AOAC method 955.04 [11]. 

### 2.4. Ascorbic Acid Content and Total Phenols

The ascorbic acid determination was performed, according to the procedure described by De Cillis et al. [3]. Briefly, 5 g of sample were placed in a centrifuge tube with 50 mL of 3% metaphosphoric acid at 4 °C. The solution was homogenized and then centrifuged for 10 min at 4000 rpm. A 0.5 mL aliquot of the supernatant was filtered with a 0.22 μm Nylon^®^ filter (Fisher Scientific^TM^ Whatman, Ma, USA). The HPLC analysis was performed using an Agilent 1100 quaternary pump and an Agilent 1260 UV detector (Agilent Thecnologies, Santa Clara, CA, USA). The chromatographic separation was performed on a Zorbax SB column (25 cm × 4.6 cm, 5 μm) with binary elution gradient, using the following solutions for the mobile phase: solution (A) KH2PO4 20 mM brought to pH 2.5 with O-phosphoric acid and solution (B) 60% methanol and 40% acetonitrile. The gradient was set according to the following program: for 2 min 5% B, from 3 to 13 min 25% B, from 14 to 18 min 90% B, from 19 to 25 min 5% B, 1 mL/min is the speed of the flow and 10 μL the volume of injection of the sample. The test was conducted by monitoring the absorbance signal at 210 nm. Peak identification and calibration was performed using the pure L-Ascorbic acid standard of Sigma-Aldrich.

For total phenols (TP), the extraction procedure reported by Renna et al. [12] was used, while the procedure described by De Cillis et al. [3] was used for TP quantification. Briefly, 0.45 g of lyophilized sample were homogenized in a methanol/water solution (80:20 *v/v*) for 1 min and then centrifuged at 6440× *g* for 5 min at 4 °C. On each extract the soluble phenol content was determined by using a colorimetric method. A total of 250 μL of Folin-ciocalteu solution and 750 μL of 7% NaCO_3_, placed on vortex for 30 s and incubated for 8 min at room temperature were added to 50 μL of the sample and 3 mL of distilled water. Then, 950 μL of distilled water were added to the mixture and left at room temperature for 2 h in the dark. The absorbance was measured at 765 nm with T60U spectrophotometer (PG Instruments, Leicestershire, UK) and results were expressed as mg of gallic acid equivalents (GAE) per 100 g FW.

### 2.5. Chlorophylls and Carotenoid Content

The content of chlorophylls and total carotenoids was determined according to the method described by Albanese et al. [13], by using a T60U spectrophotometer (PG Instruments, Leicestershire, UK). Briefly, 2 g of sample were homogenized in 10 mL of acetone/water (80:20 *v/v*) and then centrifuged at 4000× *g*. The absorbance of the extract was measured at 646, 663 and 470 nm. 

### 2.6. Content of Vicine and L-dopa

**Figure 1:**
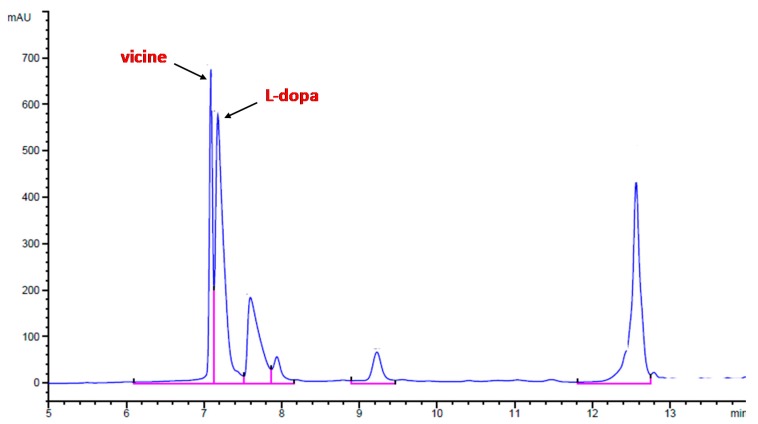
Example of chromatogram obtained after HPLC analysis for the determination of vicine and L-dopa contents.

Vicine and L-dopa content were determined according to the procedure described by De Cillis et al. [3]. Briefly, 5 g of sample were extracted with 50 mL of perchloric acid (5% *w/v*) by homogenizing for 5 min at 4 °C. The extract was centrifuged and filtered through a 0.22 μm filter (Millipore Co., Bedford, MA, USA) to remove any suspended material. For the HPLC analysis a chromatograph (Agilent 1100 quaternary pump) equipped with a UV-visible detector (Agilent 1260) at 280 nm was used. The chromatographic conditions were as follows: column C18 (Supelcosil LC, 250 × 4.6 mm, 5 μm, Supelco) with binary elution gradient at 1.0 mL/min, based on water (H2O) and acetonitrile (ACN), both containing 0.1% (*v/v*) of formic acid. The program adopted for the separation was: 0–2 min isocratic 0% ACN; 2–3 min from 0 to 10% ACN; 3–8 min isocratic at 10% ACN; 8–10 min from 10% to 90% ACN; 10–15 min at 90% ACN; 15–20 return to 0% ACN. An example of chromatogram obtained is reported in Figure 1.

### 2.7. Statistical Analysis

The two-way analysis of variance (ANOVA) was performed by using the GLM procedure (SAS software, Version 9.1) and applying a split-plot design with plant density and genotype as main factors. The Student–Newman–Keuls (SNK) test was carried out to obtain the separation of means.

The principal component analysis (PCA) was performed by using the PRINCOMP procedure (SAS software, Cary, NC, USA). The data matrix fed to PCA was made up of 12 observations—2 plant density (high and low) × 6 genotypes (Extra-early purple, Aguadulce supersimonia, FV5, Iambola, San Francesco and Cegliese)—and 19 variables (L, a*, b*, h°, C, dry matter, whole pods yield, percentage of hulls per pod, TP, titratable acidity, TSS, pH, proteins content, ascorbic acid, chlorophyll A and B, carotenoids, vicine and L-dopa,). 

## 3. Results

For all the measured parameters, the ANOVA reveals that the interaction between plant density and genotype was not significant.

### 3.1. Yield and Colour Traits

Regarding the yield of whole pods, in all cases no significant differences were found between genotypes (Table 1). At the same time, by using the high plant density (4.16 plants m^−2^) both the weight and the number of pods per unit of cultivated area were respectively 30.4 and 29.7% higher than the low density. On the other hand, independently of the plant density, Aguadulce supersimonia showed the highest value of mean pod weight, which resulted 30% higher than Cegliese and 14% than all other genotypes (Table 1). At the same time, FV5 showed a percentage of hulls higher of about 5% than Extra-early purple without any differences in comparison with other genotypes (Table 1).

L, a*, b* and C values were, respectively, 52.7, −17.5, 34.5 and 38.7 without any differences between plant densities and genotypes (Table 2). Independently of the plant density, the h° value in Cegliese was higher of about 0.5–1.0 unit than in Extra-early purple, Iambola and San Francesco (Table 2). 

### 3.2. Chemical Traits

The pH value in Aguadulce supersimonia was 5% higher than Extra-early purple, Cegliese and Iambola, while no differences were found between Aguadulce supersimonia and other genotypes (Table 3). The value of TSS, titratable acidity, DW and protein content were, respectively, 7.4 (°Brix), 0.25, 13.7 and 2.64 g 100 g^−1^ FW, without any differences between plant density and genotypes (Table 3).

As for the content of chlorophylls, total carotenoids, ascorbic acid, total phenols, vicine and L-dopa, in no case did plant density affect the values (Table 4). The chlorophyll a content was 75% higher in Cegliese than in Extra-early purple and FV5, while no differences were found between Cegliese and other genotypes. The content of chlorophyll b in Cegliese was higher of about 60% than all other genotypes with the exception of Aguadulce supersimonia (Table 4). Cegliese showed also a total carotenoid content 53% higher than in Extra-early purple and FV5, while no differences were found between Cegliese and other genotypes. As for the ascorbic acid, Extra-early purple and San Francesco showed a content higher of about 78% than all other genotypes (Table 4). The average content of vicine and L-dopa was, respectively, of 12.4 and 170.7 mg 100 g^−1^ FW, without any differences between genotypes (Table 4).

### 3.3. Principal Component Analysis

**Figure 2:**
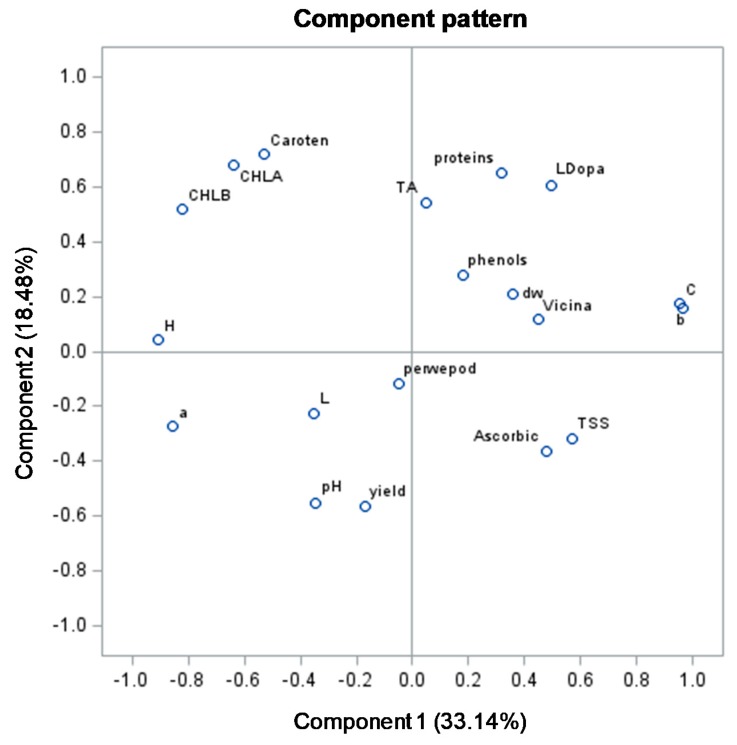
Loading plot for principal components 1 and 2, describing variation in physical and chemical parameters of six fava pod genotypes as unconventional vegetables at two plant density. L, a, b, C and h denote L, a*, b*, C and h°, respectively as colour parameters; dw, dry weight; TA, titratable acidity; TSS, total soluble content; Caroten, total carotenoids; phenols, total phenols; proteins, proteins content; CHL, chlorophyll; Ascorbic, ascorbic acid; yield, whole pods yield; perwepod, percentage of hulls per pod.

The first five principal components explained 88% of the total variance, with principal component 1 (PC1) and principal component 2 (PC2) accounting for 33.1 and 18.5%, respectively (Figure 2). PC1 was mainly correlated by colour parameters (positively by b* and C, and negatively by h°, a* and L) as well as by TTS, L-dopa, vicine and ascorbic acid (positively) and by chlorophylls, total carotenoids and pH (negatively—Figure 2). PC2 was influenced mainly by total carotenoids, chlorophylls, titratable acidity, proteins content and L-dopa (positively) and by yield and pH (negatively—Figure 2).

**Figure 3:**
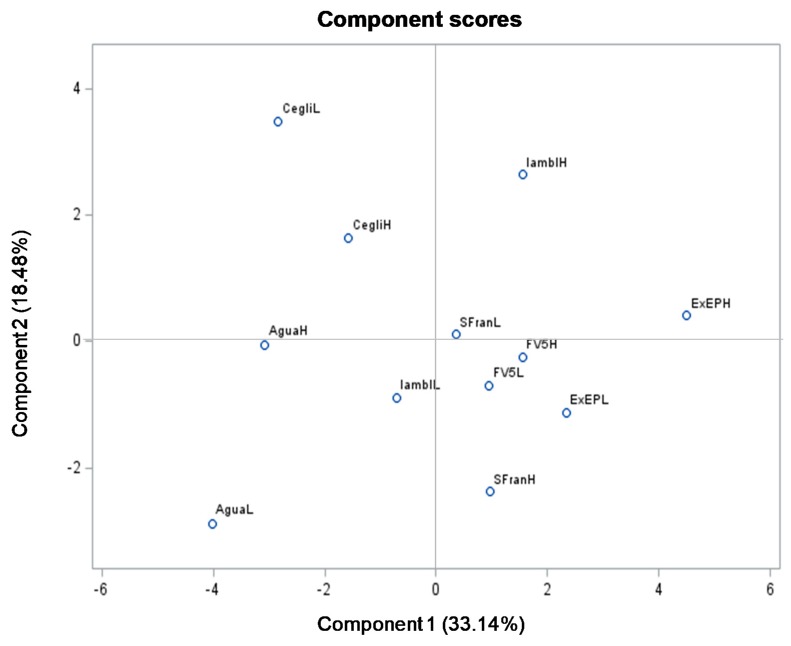
Score plot for principal components 1 and 2, describing variation in physical and chemical parameters of six fava pods genotypes as unconventional vegetables at two plant density. For each genotype, letters H and L indicate high and low plant density, respectively. SFran, San Francesco; ExEP, Extra-early purple; Iambl, Iambola; Agua, Aguadulce supersimonia; Cegli, Cegliese.

The product distribution in the score plot (Figure 3) revealed a separation between Cegliese and all other genotypes, the former located on the positive side of PC2 and negative side of PC1. Similarly, the score plot revealed a separation between Aguadulce supersimonia and all other genotypes, the former located on the negative side of both PC1 and PC2 (Figure 3). Moreover, regarding the commercial varieties there was an evident distinction between Extra-early purple and Aguadulce supersimonia, since the former was the only commercial variety located on the positive side of PC1 (Figure 3).

## 4. Discussion

In this study, for the first time, some nutritional traits of faba hulls from fresh pods were assessed in order to evaluate the potential of this unconventional vegetable as a new functional food. We compared several *Vicia faba* L. genotypes and two different plant densities to analyse also the effects of agrobiodiversity and growth conditions on the content of some nutritional and bioactive compounds. Results suggest that a great part of variation was almost exclusively due to genotypes, while only in a few cases we found differences due to the plant density. From a yield point of view, we observed that, independently of the genotype, the higher the plant density the higher was the pod yield per unit area. At the same time, the percentage of hulls was not affected by plant density, while only small differences were found between genotypes highlighting an average value of 75%. These results confirm the great benefit of considering faba hulls as unconventional vegetables instead of as a by-product, since they represent about three-quarters of the whole faba pod production.

Colour can be considered as an important quality trait of food products since it may influence the choice of consumers. Regarding vegetables, colour is associated with the change in plant tissue from green to other colours, as a consequence of chlorophylls break down and accumulation of other pigments. In this context, chlorophylls concentration in vegetable tissue can be the main factor which affects the colour in fava hulls. In our study, we found a higher h° (hue) value for Cegliese in comparison with Extra-early purple, independently of the plant density (Table 2). Hue value can be defined as a qualitative characteristic according to which colours are described as red, yellow, green, etc. More specifically, an h° value of 90 corresponds to yellow, while a value of 180 corresponds to green. In our study, the h° value ranges between 116 and 117 (Table 2), thus faba hull hues can be described as between yellow and greenish. It is important to highlight that the h° value trend was similar to the one of chlorophylls content, because Cegliese showed a higher value than Extra-early purple (Table 4). Therefore, the lower h° value in Extra-early purple indicates a hue which can be described as less green than Cegliese, probably due to its lower chlorophylls content. The trend for the h° value and chlorophylls content was confirmed by PCA, since h° and chlorophylls (a and b) were located on the upper left quadrant (Figure 2). At the same time, Cegliese at high and low density, both located on the same quadrant, were positively correlated with h° and chlorophylls, while Extra-early purple at low and high density, both located to the right in PC1 plot far from the origin, were inversely correlated with h° and chlorophylls (Figure 3).

From a nutritional point of view, faba hulls show an average protein content of about 2.6 g 100 g^−1^ FW, without any differences between plant densities and genotypes. In a study aimed to evaluate the quality of immature fresh seeds in some landraces and commercial varieties of *Vicia faba* L., De Cillis et al. [3] found an average protein content of about 5.1 g 100 g^−1^ FW, while the United States Department of Agriculture (USDA) reports a protein content of 5.6 g 100 g^−1^ FW in immature seeds of faba beans [14], suggesting faba seeds contain higher protein amounts than the hulls. On the other hand, it should be considered that faba hulls contain a protein amount slightly higher than fresh pods of other legumes such as snap beans, since their average protein content is of about 1.8 g 100 g^−1^ FW [15,16]. 

Ascorbic acid content was, on average, 40 mg 100 g^−1^ FW, ranging from 30 to 58 mg 100 g^−1^ FW between genotypes (Table 4). This vitamin is essential for humans, considering its numerous functions for human health [17]. Ascorbic acid content in fava hulls appears tens of times lower than ones reported by De Cillis et al. [3] in immature seeds of faba beans (on average, 545 mg 100 g^−1^ FW). At the same time, ascorbic acid content in faba hulls seems to be similar in comparison with one reported for immature seeds of faba beans (33 mg 100 g^−1^ FW) [14]. Furthermore, it should be considered that faba hulls contain an ascorbic acid content at least two times higher than fresh pods of other legumes such as snap beans, since their average content of ascorbic acid is of about 14 mg 100 g^−1^ FW [15,16]. Therefore, our results suggest that faba hulls can be judged a good source of ascorbic acid, considering its recommended daily intake of 45–70 mg [18]. 

Phenols represent an important group of antioxidants for humans. In our study the total phenols content was significantly different among the genotypes, ranging from 443 to 646 mg 100 g^−1^ FW (Table 4). Although the literature lacks information regarding the content of phenols in faba hulls, it is interesting to note that De Cillis et al. [3] found an average total phenols content of 551 mg 100 g^−1^ FW in immature fresh seeds of faba bean. On the other hand, other authors [19] found an amount of about 49 mg 100 g^−1^ (on dry weight basis). Thus, our results highlight the high amount of total phenols in fresh faba products, not only in seeds but also in hulls. To this end, it could be interesting to compare also the total phenols content between faba hulls and other common vegetables. In a study aimed to evaluate the phenols content in 23 vegetables, Vinson et al. [20] found the highest content (916 mg 100 g^−1^ FW) in kidney beans followed by pinto beans (829 mg 100 g^−1^ FW), garlic (374 mg 100 g^−1^ FW), beet (236 mg 100 g^−1^ FW), corn (142 mg 100 g^−1^ FW) and broccoli (105 mg 100 g^−1^ FW). In all other cases (asparagus, snap bean, kidney bean, cabbage, carrot, cauliflower, celery, cucumber. lettuce, mushroom, red and yellow onion, bell pepper, potato, green squash, spinach, sweet potato and tomato), the phenols content was lower than 100 mg 100 g^−1^ FW. Therefore, the results of the present study show that faba hulls can contain an amount of phenols at least five times higher than several other common vegetables hence suggesting their consumption for the recommended intake of this important group of antioxidants.

Vicine is a pyrimidine β-glycoside present in the cotyledons of the faba bean, which is hydrolysed by the G6PD. This glycoside is a potential favism-inducing factor, since it can cause acute haemolysis in subjects affected by a deficiency of this enzyme due to a gene mutation. It is important to note that people affected by favism are always G6PD deficient, while not all people with a G6PD deficiency develop haemolysis after fava beans ingestion. Therefore, G6PD deficiency can be considered as a necessary cause of favism although it is a not sufficient cause. Moreover, the great majority of favism manifestations occurs in people with severely deficient variants of G6PD, while only in a few cases this disease has been observed in subjects with other G6PD variants [21]. Although convicine can cause the same problems in subjects affected by β-glucosidases deficiency, we decided to determinate only vicine content, considering that in *Vicia faba* L. convicine content is about half the vicine one [22,23]. In our study, the average content of vicine was 12.4 mg 100 g^−1^ FW, without any difference between genotypes and plant density (Table 4). De Cillis et al. [3] reported a vicine content over 2000 mg 100 g^−1^ DW in immature seeds of faba beans, while other authors [24] reported a content ranging between 450 and 900 100 g^−1^ DW. Our results highlight the much lower content of vicine in faba hulls (90 mg 100 g^−1^ DW, on average) instead of faba seed, suggesting this unconventional vegetable as an alternative instead of faba seed for G6PD deficient subjects.

L-dopa, a precursor of dopamine, is considered a very important compound currently used to increase dopamine concentrations in patients affected by Parkinson’s disease and dopamine-responsive dystonia [4]. It is well known that faba beans can be considered a natural source of L-dopa with an amount able to be pharmacologically active on subjects affected by Parkinson’s disease [25]. Actually, some authors reported that the use of fresh fava beans as meal supplementation is effective as a means of reducing the levodopa off-period disability in patients with Parkinson’s disease [26]. Other authors reported that in patients with Parkinson’s disease who consumed fresh fava beans, the plasma L-dopa levels remain at higher levels for longer periods than in healthy subjects [27]. At the same time, it should be considered that high plasma levels of L-dopa gained through the consumption of faba beans are comparable to the ones obtained by using synthetic L-dopa preparations [28]. It is important to highlight that the increase of the L-dopa plasma level caused by ingestion of faba beans can improve motor symptoms without side effects such as intense dyskinesia [4]. Nevertheless, L-dopa concentration in faba beans may vary markedly based on genotype, plant density, environment conditions, as well as several other factors. Although the literature lacks information regarding the content of L-dopa in faba hulls, it is interesting to note that De Cillis et al. [3] found a L-dopa content in fresh faba seeds ranging from 2.6 to 10.1 mg 100 g^−1^ FW, while other authors [29] reported an amount of about 50 mg 100 g^−1^ FW. In the present study L-dopa was quantified in faba hulls, and we found an average content of about 170 mg 100 g^−1^ FW, without differences between genotypes and plant densities (Table 4). Therefore, our results highlight the very high content of L-dopa in faba hulls, suggesting their consumption as a suitable foodstuff for patients with Parkinson’s disease.

## 5. Conclusions

In this study, for the first time, some nutritional traits of faba hulls from fresh pods of six genotypes were assessed in order to evaluate the potential of this unconventional vegetable as a new functional food. From a yield point of view, our results highlight that faba hulls represent about 75% of the total fava bean yield from the field, suggesting the great advantage of considering these plant parts as unconventional vegetables instead of a by-product. As for the nutritional traits, all genotypes showed a low content of the antinutritional factor vicine, and a very high content of phenols and L-dopa, a dopamine precursor used for Parkinson’s disease treatment. In conclusion, this study highlights the good potential of faba hulls as an unconventional vegetable, suggesting its use as a new functional food in the daily diet and also for patients with Parkinson’s disease. Future research activities may be aimed to assess the use of faba hulls as a meal supplementation in patients with Parkinson’s disease through clinical trials, as well as the possibility of processing hull-based food products with standardized L-dopa content.

## Figures and Tables

**Table 1:** Main effects of plant density and genotypes on cumulative whole pod production and percentage of hulls. For each parameter are reported mean values ± standard deviation.

	Cumulative Whole Pods Yield		Mean Pod Weight	Hulls
	(Number m^−2^)	(g m^−2^)	(g)	(%)
**Density (Plants m^−2^)**				
4.16	86.3 ± 16.0	1794 ± 373	20.7 ± 1.7	74.7 ± 4.6
2.08	66.5 ± 16.5	1376 ± 403	20.6 ± 2.4	74.5 ± 4.6
**Genotype**				
Aguadulce supersimonia	78.6 ± 23.0	1839 ± 510	23.5 ± 2.1 a	74.0 ± 4.5 ab
Extra-early purple	86.4 ± 22.1	1737 ± 502	20.0 ± 1.0 b	73.1 ± 5.9 b
Cegliese	70.1 ± 23.7	1275 ± 487	18.0 ± 1.1 c	73.7 ± 4.7 ab
FV5	73.0 ± 15.3	1529 ± 352	20.9 ± 0.7 b	76.8 ± 4.0 a
Iambola	73.0 ± 11.4	1502 ± 309	20.4 ± 1.4 b	74.5 ± 4.2 ab
San Francesco	77.4 ± 18.8	1627 ± 372	21.1 ± 0.8 b	75.6 ± 3.8 ab
***Significance***				
Density	*	*	ns	ns
Genotype	ns	ns	***	*
Density*Genotype	ns	ns	ns	ns

Significance: ns = not significant; * significant for *p* ≤ 0.05. Different letters indicate statistically significant differences at *p* = 0.05. Harvesting dates: 10/4/2018, 18/4/2018, 24/4/2018, 5/4/2018.

**Table 2:** Main effects of plant density and genotypes on CIE*Lab* colour traits (L, a*, b*, h° and C) of fava hulls as unconventional vegetable. For each parameter are reported mean values ± standard deviation.

	L	a*	b*	h°	C
**Density (Plants m^−2^)**					
4.16	52.9 ± 2.1	−17.7 ± 0.8	34.8 ± 1.4	117.0 ± 0.6	39.0 ± 1.6
2.08	52.6 ± 2.2	−17.4 ± 0.7	34.2 ± 1.2	117.0 ± 0.7	38.4 ± 1.3
**Genotype**					
Aguadulce supersimonia	53.4 ± 2.8	−17.2 ± 0.8	33.7 ± 1.3	117.1 ± 0.5 ab	37.8 ± 1.5
Extra-early purple	53.9 ± 2.5	−17.3 ± 0.8	35.0 ± 1.3	116.4 ± 0.7 c	39.0 ± 1.5
Cegliese	52.1 ± 2.3	−17.9 ± 1.1	34.3 ± 1.6	117.5 ± 0.6 a	38.7 ± 1.9
FV5	51.9 ± 1.2	−17.8 ± 0.6	34.7 ± 1.2	117.2 ± 0.5 ab	39.1 ± 1.3
Iambola	52.6 ± 1.8	−17.4 ± 0.6	34.7 ± 1.3	116.7 ± 0.4 bc	38.8 ± 1.4
San Francesco	52.5 ± 1.8	−17.6 ± 0.5	34.6 ± 1.2	116.9 ± 0.4 b	38.9 ± 1.2
***Significance***					
Density	ns	ns	ns	ns	ns
Genotype	ns	ns	ns	**	ns
Density*Genotype	ns	ns	ns	ns	ns

Significance: ns = not significant; ** significant for *p* ≤ 0.01. Different letters indicate statistically significant differences at *p* = 0.05.

**Table 3:** Main effects of plant density and genotypes on pH, total soluble solids (TSS) titratable acidity (TA), dry weight and protein content of fava hulls as unconventional vegetable. For each parameter are reported mean values ± standard deviation.

	pH	TSS	TA	Dry Weight	Protein
		(°Brix)	(g 100 g^−1^ FW)		
**Density (Plants m^−2^)**					
4.16	5.5 ± 0.1	7.5 ± 1.1	0.25 ± 0.05	13.5 ± 1.0	2.63 ± 0.30
2.08	5.5 ± 0.1	7.3 ± 0.9	0.26 ± 0.06	14.0 ± 1.8	2.66 ± 0.34
**Genotype**					
Aguadulce Supersimonia	5.7 ± 0.1 a	6.9 ± 0.6	0.24 ± 0.06	13.1 ± 0.7	2.46 ± 0.20
Extra-early purple	5.4 ± 0.1 b	7.9 ± 0.9	0.25 ± 0.05	14.9 ± 1.5	2.80 ± 0.42
Cegliese	5.4 ± 0.2 b	6.8 ± 1.4	0.26 ± 0.08	13.9 ± 0.7	2.79 ± 0.32
FV5	5.5 ± 0.1 ab	7.3 ± 0.7	0.22 ± 0.04	13.8 ± 2.4	2.64 ± 0.37
Iambola	5.4 ± 0.2 b	7.7 ± 1.0	0.29 ± 0.02	14.1 ± 0.8	2.75 ± 0.15
San Francesco	5.5 ± 0.1 ab	7.9 ± 0.7	0.26 ± 0.06	12.8 ± 1.3	2.44 ± 0.27
***Significance***					
Density	ns	ns	ns	ns	ns
Genotype	**	ns	ns	ns	ns
Density*Genotype	ns	ns	ns	ns	ns

Significance: ns = not significant; ** significant for *p* ≤ 0.01. Different letters indicate statistically significant differences at *p* = 0.05.

**Table 4:** Main effects of plant density and genotypes on the content of chlorophyll (Chl) a and b, total carotenoids (TC), ascorbic acid, total phenols and vicine and L-dopa of fava pods as unconventional vegetable. Total phenols content is expressed as gallic acid equivalent. For each parameter are reported mean values ± standard deviation.

	Chl a	Chl b	TC	Ascorbic Acid	Total Phenols	Vicine	L-Dopa
	(µg g^−1^ FW)			(mg 100 g^−1^ FW)			
**Density (Plants m^−2^)**							
4.16	17.0 ± 5.4	6.7 ± 2.7	7.2 ± 1.8	42 ± 18	563 ± 129	14.5 ± 5.3	178.4 ± 32.2
2.08	16.2 ± 4.9	7.2 ± 1.9	7.0 ± 1.9	37 ± 10	602 ± 114	10.4 ± 3.6	163.1 ± 27.3
**Variety**							
Aguadulce Supersimonia	18.1 ± 5.2 ab	8.3 ± 1.8 ab	7.5 ± 1.5 ab	32 ± 3 b	443 ± 70 b	13.1 ± 7.8	158.5 ± 20.2
Extra-early purple	12.8 ± 3.1 b	4.5 ± 2.6 c	6.0 ± 1.4 b	53 ± 7 a	546 ± 113 ab	14.6 ± 6.8	185.5 ± 33.7
Cegliese	22.8 ± 5.4 a	9.6 ± 2.0 a	9.1 ± 2.3 a	33 ± 2 b	638 ± 50 a	10.4 ± 3.4	176.3 ± 21.6
FV5	13.3 ± 3.0 b	6.2 ± 0.9 bc	5.9 ± 1.2 b	30 ± 3 b	570 ± 120 ab	13.7 ± 4.5	182.8 ± 28.5
Iambola	16.3 ± 4.2 ab	6.9 ± 1.1 bc	7.1 ± 1.3 ab	30 ± 3 b	649 ± 77 a	10.3 ± 3.4	165.7 ± 44.7
San Francesco	16.2 ± 3.7 ab	6.3 ± 1.7 bc	7.2 ± 1.7 ab	58 ± 20 a	651151 a	12.6 ± 1.7	155.7 ± 26.4
***Significance***							
Density	ns	ns	ns	ns	ns	ns	ns
Genotype	**	***	**	***	**	ns	ns
Density*Genotype	ns	ns	ns	ns	ns	ns	ns

Significance: ns = not significant; ** and *** significant for *p* ≤ 0.01 and *p* ≤ 0.001, respectively. Different letters indicate statistically significant differences at *p* = 0.05.
